# Carrier-mediated ferromagnetism in the magnetic topological insulator Cr-doped (Sb,Bi)_2_Te_3_

**DOI:** 10.1038/ncomms9913

**Published:** 2015-11-19

**Authors:** Mao Ye, Wei Li, Siyuan Zhu, Yukiharu Takeda, Yuji Saitoh, Jiajia Wang, Hong Pan, Munisa Nurmamat, Kazuki Sumida, Fuhao Ji, Zhen Liu, Haifeng Yang, Zhengtai Liu, Dawei Shen, Akio Kimura, Shan Qiao, Xiaoming Xie

**Affiliations:** 1 State Key Laboratoryof Functional Materials for Informatics, Shanghai Institute of Microsystem and Information Technology, Chinese Academy of Sciences, 865 Chang Ning Road, Shanghai 200050, China; 2 CAS-Shanghai Science Research Center, 239 Zhang Heng Road, Shanghai 201203, China; 3 Graduate School of Science, Hiroshima University, 1-3-1 Kagamiyama, Higashi-Hiroshima 739-8526, Japan; 4 Condensed Matter Science Division, Quantum Beam Science Center, Japan Atomic Energy Agency, Sayo, Hyogo 679-5148, Japan; 5 School of physical science and technology, ShanghaiTech University, 319 Yueyang Road, Shanghai 200031, China; 6 Department of Physics, State Key Laboratory of Surface Physics, and Laboratory of Advanced Materials, Fudan University, Shanghai 200433, China

## Abstract

Magnetically doped topological insulators, possessing an energy gap created at the Dirac point through time-reversal-symmetry breaking, are predicted to exhibit exotic phenomena including the quantized anomalous Hall effect and a dissipationless transport, which facilitate the development of low-power-consumption devices using electron spins. Although several candidates of magnetically doped topological insulators were demonstrated to show long-range magnetic order, the realization of the quantized anomalous Hall effect is so far restricted to the Cr-doped (Sb,Bi)_2_Te_3_ system at extremely low temperature; however, the microscopic origin of its ferromagnetism is poorly understood. Here we present an element-resolved study for Cr-doped (Sb,Bi)_2_Te_3_ using X-ray magnetic circular dichroism to unambiguously show that the long-range magnetic order is mediated by the *p*-hole carriers of the host lattice, and the interaction between the Sb(Te) *p* and Cr *d* states is crucial. Our results are important for material engineering in realizing the quantized anomalous Hall effect at higher temperatures.

## Abstract (web summary)

Magnetically doped topological insulators may exhibit exotic transport phenomena such as the quantum anomalous Hall effect, however the underlying mechanisms of ferromagnetic order are currently debated. Here, the authors reveal stabilized ferromagnetism in Cr-doped (Sb,Bi)_2_Te_3_ mediated by Te and Sb *p*-hole carriers.

## Graphical abstract

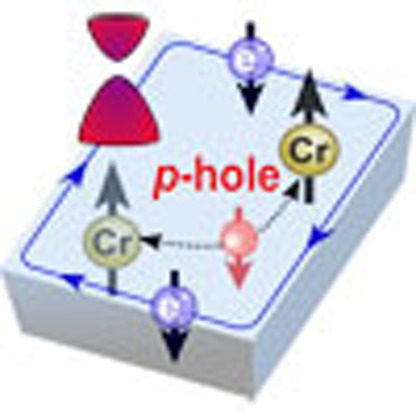

The experimental observation of the quantum anomalous Hall (QAH) effect^1^,^2^,^3^, a hallmark of topologically non-trivial states in magnetic topological insulators (TIs)^4^,^5^,^6^,^7^,^8^,^9^,^10^,^11^, has stimulated unprecedented research activities in the field of materials showing topologically protected surface states. While the origins of such strongly coupled magnetism in TIs are still under debate, Checkelsky *et al.*^12^ reported the suppression of ferromagnetism in Mn-doped Bi_2_Te_3−*y*_Se_*y*_ by increasing carrier densities, suggesting a Dirac-fermion-mediated origin for the surface ferromagnetism in TIs. In contrast, for the long-range ferromagnetic order in a Cr-doped (Bi_*y*_Sb_1−*y*_)_2_Te_3_ film (0≤*y*≤0.5), Chang *et al.*^13^ demonstrated an independence of Curie temperature (*T*_C_) with carrier density, typically ∼30–35K. To settle this conflict on the role of carriers in magnetic TIs, the microscopic origin of this magnetism needs to be studied systematically for various *T*_C_, rather than simply chasing a high one. One should certainly be guided by the analogy with conventional dilute magnetic semiconductors where extrinsic magnetism, for instance, from the clustering of a magnetic dopant, also gives rise to the elevation of the *T*_C_. Indeed, the aggregation of Cr dopants in Bi_2_Se_3_, which resulted in an energy gap opening for Dirac surface states even without long-range magnetic order, has been reported very recently^14^. By spin-polarized scanning tunnelling microscopy, Yang *et al.*^15^ revealed in Cr_0.05_Sb_1.95_Te_3_ that the spin polarization of the surface states lies in the surface plane, and deviates from the bulk states being oriented along the out-of-plane easy axis. These results suggest that the surface magnetism in the Cr-doped TIs might not simply follow the bulk one. Since the observation of the QAH effect with Cr-doped (Sb,Bi)_2_Te_3_ system is restricted <100 mK (refs 1, 2, 3), the development of ferromagnetic TIs with much higher *T*_C_ are strongly desired. Apart from the complex surface magnetism, to raise the bulk *T*_C_ would lead to a better stabilization of surface ferromagnetism. Therefore, the first important step in realizing the QAH at higher temperature would be to investigate the driving mechanism of ferromagnetism.

In this work, we identify the element-resolved magnetism in the Cr-doped magnetic TI (Sb,Bi)_2_Te_3_ using X-ray magnetic circular dichroism (XMCD) combined with photoemission spectroscopy and a first-principles calculation. We find that, with increasing Cr concentration, the bulk ferromagnetism is more stabilized in the Cr-doped (Sb,Bi)_2_Te_3_ system. More importantly, we have detected the magnetic moments not only for the Cr dopant *d* states but also for the Sb and Te *p* states in the host lattice; they are found to be absent on the Bi-site, suggesting that the formation of long-range magnetic order is mainly mediated by both Te and Sb *p*-hole carriers.

## Results

### Carrier density and magnetism of Cr_*x*_(Sb_1−*y*_Bi_*y*_)_2−*x*_Te_3_

**Figure 1:**
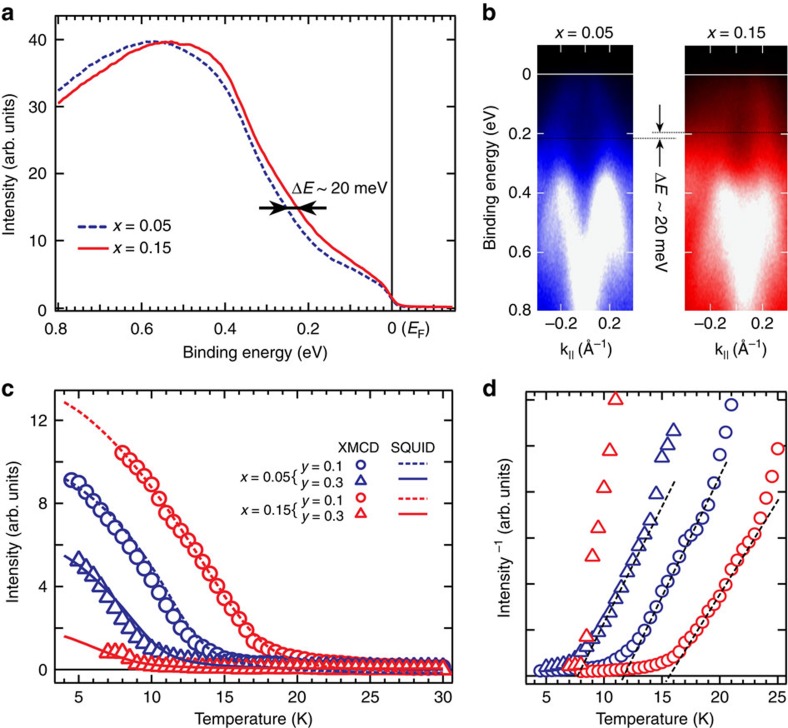
Carrier dependence of the Curie temperature in Cr-doped (Sb,Bi)_2_Te_3_ samples. (**a**) Angle-integrated photoemission spectra of Cr_*x*_(Sb_0.9_Bi_0.1_)_2−x_Te_3_ (*x*=0.05 and 0.15) measured at 70 K with 21.2 eV photons. (**b**) Angle-resolved photoemission spectra along 
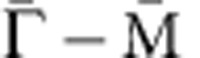
 direction of the surface Brillouin zones of Cr_*x*_(Sb_0.9_Bi_0.1_)_2−x_Te_3_ (*x*=0.05 and 0.15). (**c**) Magnetization curves against temperature (*M*–*T*) for Cr_*x*_(Sb_1−*y*_Bi_*y*_)_2−*x*_Te_3_ (*x*=0.05 and 0.15; *y*=0.1 and 0.3) obtained by plotting the X-ray magnetic circular dichroism (XMCD) intensity at the Cr L_3_ edge (*hν*=575.3 eV), compared with *M*–*T* obtained by superconducting quantum interference device measurement with applied magnetic field of 0.1 T; (**d**) *T*_C_ estimates of Cr_*x*_(Sb_1−*y*_Bi_*y*_)_2−*x*_Te_3_ obtained by plotting the inverse of XMCD intensity at the Cr L_3_ edge against temperature for *x*=0.05 (blue) and 0.15 (red), and *y*=0.1 (circle) and 0.3 (triangle). The black dashed lines are guide for eyes.

To directly visualize the changes in carrier density on Cr-doping, photoemission spectroscopy measurements were performed on samples with different Cr concentrations, all of which were carefully examined with X-ray diffraction and Magnetic Property Measurement System (Quantum Design) using a superconducting quantum interference device (SQUID) for crystalline quality and magnetic properties prior to photoemission experiments and following XMCD measurements (Supplementary Figs 1 and 2 and Supplementary Methods). Figure 1a shows the angle-integrated photoemission spectra of Cr_*x*_(Sb_0.9_Bi_0.1_)_2−*x*_Te_3_ (*x*=0.05 and 0.15). The corresponding angle-resolved photoemission spectra (ARPES) taken along the 
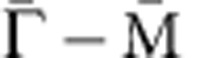
 direction of the surface Brillouin zones are shown in Fig. 1b. The observed dispersive bands approaching the Fermi energy are ascribed to the bulk components instead of the surface Dirac cones, suggesting a bulk metallic feature for these samples. The spectrum of the *x*=0.15 sample (red solid line in Fig. 1a) shifts towards lower binding energy by *ΔE*∼20 meV with respect to that with lower Cr concentration (*x*=0.05, blue dashed line in Fig. 1a), as marked at the onset of bulk band in Fig. 1a,b. It tells us that the introduced holes by Cr-doping behave as the host-lattice carriers and its number increases with increasing Cr concentration, being consistent with previously reported Cr-doped Bi_2_Se_3_ film^16^,^17^. On the other hand, the Bi elements substituted at the Sb-sites reduce the hole-type carrier concentration because the Sb_2_Te_3_ is originally p-type metal where the bulk valence band crosses the Fermi energy (Supplementary Fig. 3 and Supplementary Note 1).

XMCD is a powerful method that is able to probe the magnetism selectively on different elements (Supplementary Figs 4 and 5, and Supplementary Methods), which is suitable for the study on magnetically doped system, such as diluted magnetic semiconductors and magnetically doped TIs^18^,^19^. We then measured the amplitude of the XMCD signals as a function of temperature at the Cr L_3_ edge corresponding to the excitation from Cr 2*p* core electronic states to the partially occupied 3*d* orbitals^20^, which is proportional to the Cr-3*d*-spin magnetic moment of the samples (the curve is hereafter referred to as the *M*–*T* curve). The *M*–*T* curves of four Cr_*x*_(Sb_1−*y*_Bi_*y*_)_2−*x*_Te_3_ samples of different Cr and Bi concentrations were measured using XMCD and compared with those measured by SQUID (Fig. 1c). With fixed Bi concentration (*y*=0.1), the sample with higher Cr concentration (*x*=0.15, red circle) shows the highest *T*_C_=15 K, as estimated by plotting the inverse of the *M*–*T* curve shown in Fig. 1d; the *T*_C_ value is similar to the one previously reported by Chang *et al.*^1^ evidencing the QAH effect. However, with the larger Bi concentration but fixed Cr dopant concentration (*x*=0.05 or 0.15), *T*_C_ values experience an abrupt decrease (Fig. 1c,d). The suppression of *T*_C_ by Bi substitutions on the Sb-sites suggests a strong reduction in long-range magnetic order; this issue will be discussed further with our XMCD experimental results and theoretical considerations.

As XMCD method is based on x-ray absorption spectroscopy, it provides the magnetic information averaged through several nanometres from the surface in a total-electron-yield (TEY) mode adopted in the present study. We therefore further compared the *M*–*T* curves obtained through XMCD with the magnetization curves measured by a SQUID; the latter provides the total magnetic moment of the bulk crystals. Marked by solid and dashed lines in Fig. 1c, the SQUID-measured *M*–*T* curves for all four samples show excellent consistency with the XMCD results, signifying that the observed XMCD spectra in this work mostly reflect the bulk magnetic moments. This may explain why no deviation of the surface magnetism from the bulk, as reported in ref. 15, was observed in the present XMCD study.

### Element-resolved magnetic moments of Cr_*x*_(Sb_1−*y*_Bi_*y*_)_2−*x*_Te_3_

**Figure 2:**
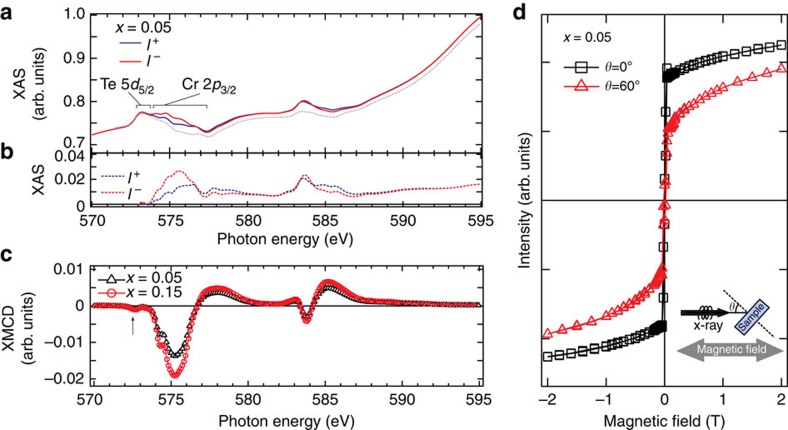
X-ray absorption spectroscopy (XAS) and X-ray magnetic circular dichroism (XMCD) spectra of Cr_*x*_(Sb_0.9_Bi_0.1_)_2−*x*_Te_3_ samples at Cr L_23_ edges. (**a**) Normalized XAS spectra of Cr_0.05_(Sb_0.9_Bi_0.1_)_1.95_Te_3_ at the Cr L_23_ edges in a magnetic field of 0.1 T measured by circularly polarized soft X-ray at 5 K. The grey dashed line indicates the spectral background from Te M_45_ edge measured on (Sb_0.5_Bi_0.5_)_2_Te_3_ sample. (**b**) XAS spectra of Cr_0.05_(Sb_0.9_Bi_0.1_)_1.95_Te_3_ at Cr L_23_ after background from Te M_45_ absorption edges were subtracted. (**c**) XMCD spectra of Cr_*x*_(Sb_0.9_Bi_0.1_)_2−*x*_Te_3_ (*x*=0.05 and 0.15) at the Cr L_23_ edges in a magnetic field of 0.1 T at 5 K, obtained by taking the difference of the normalized XAS spectra. The arrow indicates a small intensity at the energy of Te M_5_ edge. (**d**) Perpendicular magnetic anisotropy of Cr_0.05_(Sb_0.9_Bi_0.1_)_1.95_Te_3_ revealed by angle-dependent *M–H* measurement measured at 5 K. *θ* is defined as the angle between the sample surface normal direction and the incident X-ray that is always parallel (anti-parallel) to the magnetic field direction as shown in the inset.

Detailed XMCD spectra, measured element-selectively on the Cr-site below *T*_C_, are shown in Fig. 2. The circular-polarized XAS spectra (Fig. 2a) at the Cr L_23_ edges of Cr_x_(Sb_1−*y*_Bi_*y*_)_2−*x*_Te_3_ (*x*=0.05, *y*=0.1, *T*_C_∼15 K) were recorded at 5 K and 0.1 T, the intensities of which are normalized to 1 at the energy of 595 eV (see Supplementary Fig. 6 and Supplementary Methods for detailed normalization process); here the blue and red lines represent the XAS spectrum with the applied magnetic field parallel and anti-parallel, respectively, to the photon spin of the circularly polarized X-rays. Note that a weak magnetic field, 0.1 T, is applied to improve the statistics of the dichroic signals. The multi-peak structures around photon energies (*hν*) ∼575 and 585 eV result from the excitations for the Cr 2*p*_3/2_ and 2*p*_1/2_ core levels, respectively, which slightly overlap with the broad Te M_45_ (3*d*) edges. By comparing with the XAS spectrum acquired from the Cr-free sample (Sb_0.5_Bi_0.5_)_2_Te_3_ shown with grey dashed line in Fig. 2a, we can more precisely estimate the XAS intensity contributed from the Cr L_23_ edges as shown in Fig. 2b, where the XAS intensities are *I*^+^=0.014 and *I*^−^=0.027 for the two different photon polarizations, respectively. This results in a dichroism intensity of *I*^+^−*I*^−^=0.013, which is 31.7% of the total XAS intensity (*I*^+^+*I*^−^=0.041) at Cr L_3_ edge for *x*=0.05 sample. Figure 2c shows the XMCD spectra of two Cr_*x*_(Sb_0.9_Bi_0.1_)_2−*x*_Te_3_ samples with different Cr concentrations (*x*=0.05 and 0.15) obtained by taking the difference between the normalized XAS spectra for the two polarizations, which reveals the increase of Cr magnetic moment with higher Cr concentration from the increase of XMCD intensity at Cr L_23_ edges. These XAS/XMCD results also rule out the possible clustering of Cr at increased doping concentrations in our samples; otherwise, the XAS and XMCD line shapes would have been significantly modified. By measuring the angular-dependent XMCD intensity, which is proportional to the magnetization of the selected element, at the Cr L_3_ edge (575.03 eV) as a function of magnetic field (*M–H* curve, Fig. 2d), the easy magnetization axis is found to be perpendicular to the sample surface satisfying the crucial condition for the time-reversal-symmetry breaking of the topologically non-trivial states. The *M–H* curve recorded for the tilted magnetization (*θ*=60°) exhibits a much smaller XMCD intensity at 0 T, and gradually increases with magnetic field as shown in Fig. 2d, confirming a strong magnetic anisotropy preferred along the out-of-plane direction. The whole set of the *M–H* curves for samples with different Cr and Bi concentration, and the XMCD spectra measured with remnant magnetization can be found in the Supplementary Fig. 7. Moreover, an additional peak, indicated by an arrow, can be observed on the low-energy side of the Cr L_3_ edge (Fig. 2c), which was unreported in previous research on the Cr-included chalcogenides^21^,^22^. This additional peak coincides energetically with the Te M_5_ absorption edges, indicating a possible magnetic moment residing at the Te site of the host lattice.

**Figure 3:**
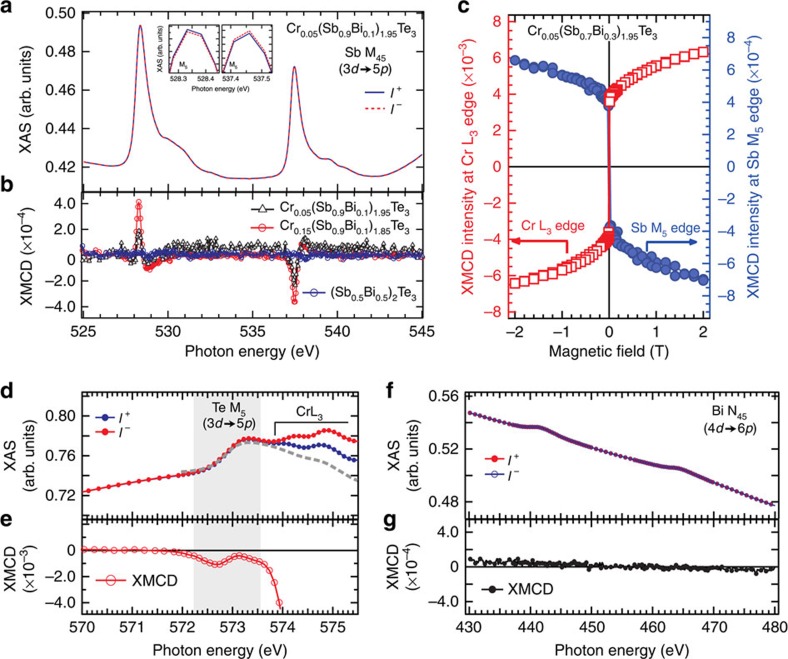
Element-resolved magnetic structures in Cr-doped (Sb,Bi)_2_Te_3_. (**a**) Normalized X-ray absorption spectroscopy (XAS) spectra of Cr_0.05_(Sb_0.9_Bi_0.1_)_1.95_Te_3_ at the Sb M_45_ edges in a magnetic field of 0.1 T measured at 5 K; (**b**) X-ray magnetic circular dichroism (XMCD) spectra of Cr_*x*_(Sb_0.9_Bi_0.1_)_2−*x*_Te_3_ (*x*=0.05 and 0.15) at Sb M_45_ edges, compared with Cr-free sample (Sb_0.5_Bi_0.5_)_2_Te_3_; (**c**) Magnetization curves as a function of magnetic field taken at Cr L_3_ edge (red squares, left axis) and Sb M_5_ edge (blue circles, right axis) of Cr_0.05_(Sb_0.7_Bi_0.3_)_1.95_Te_3_ at 5 K; (**d**,**e**) XAS and XMCD spectra of Cr_0.15_(Sb_0.9_Bi_0.1_)_1.85_Te_3_ at the Te M_5_ edge, compared with the XAS spectrum of (Sb_0.5_Bi_0.5_)_2_Te_3_ (grey dashed line); (**f**,**g**) Normalized XAS and XMCD spectra of Cr_0.15_(Sb_0.9_Bi_0.1_)_1.85_Te_3_ at the Bi N_45_ edge taken at higher magnetic field, 1 T at 5 K.

To examine the possible induced magnetic moments at the non-magnetic elements in the host lattice, we measured the XMCD spectra at the Sb absorption edges. Figure 3a shows the circular-polarized XAS spectra of Cr_0.05_(Sb_0.9_Bi_0.1_)_1.95_Te_3_. Even though only a very slight difference can be seen in these normalized XAS spectra, the magnified views (see insets) show a clear reversal of the XAS intensities between the Sb M_5_ (528.3 eV) and M_4_ (537.4 eV) edges. One can also find that the dichroism signal at Sb M_5_ edge (*I*^+^−*I*^−^=2 × 10^−4^ as shown in Fig. 3b) is only 0.14% of the total XAS intensity (*I*^+^+*I*^−^=0.146 estimated from the edge-jump of the Sb M_5_ absorption edge), which is 2 orders of magnitude smaller than that at the Cr L_3_ edge (31.7%). For Cr_0.15_(Sb_0.9_Bi_0.1_)_1.85_Te_3_ sample with higher Cr concentration, the XMCD spectrum exhibits higher intensity at Sb M_45_ edges (Fig. 3b) than that for Cr_0.05_(Sb_0.9_Bi_0.1_)_1.95_Te_3_ sample, following the change at the Cr L_23_ edges. The XMCD signals in the same energy range for the Cr-free sample, (Sb_0.5_Bi_0.5_)_2_Te_3_ (*x*=0, *y*=0.5), is given for comparison (blue circle in Fig. 3b). Clearly, the Cr-free sample shows negligible XMCD intensity over the same energy range, confirming that the tiny XMCD signals captured at Sb M_45_ edges originate from the intrinsic magnetism rather than any instrumental asymmetry. The similar intensity with opposite signs of the XMCD signals observed at M_4_ and M_5_ edges suggests that the spin origin dominates the magnetic moment on Sb-site. Figure 3c shows the element-specific *M–H* curves at the Sb and Cr edges measured at 5 K. Nonzero intensities are seen at zero magnetic field for both edges. More importantly, having considered that the Sb M_45_ absorption edges are related to the *d→p* transition, whereas the Cr L_23_ edges correspond to the *p→d* transition, the opposite sign for these edges indicates a parallel coupling between the Sb 5*p* and Cr 3*d* spins^23^. Although XMCD signal at Sb M_5_ edge shows opposite sign compared with Cr L_3_ edge, the field-dependent evolution of the XMCD signal magnitude at Sb M_5_ edge is almost the same as that of Cr L_3_ edge, further confirming a parallel coupling between the magnetic moments of Sb and Cr.

Returning to the unexpected XMCD intensity at the pre-edge of Cr L_3_ (Fig. 2c), a magnified view in this energy region (Fig. 3d,e) is compared with the XAS spectrum of the Cr-free sample. The observed XMCD intensity (*hν*=572.5 eV) overlaps energetically with the Te M_5_ edge, indicating an induced magnetic moment at the Te site in the Cr-doped samples. More interestingly, in analogy with the Sb edge (Fig. 3a,b), when the sign of the XMCD signal at the Te M_5_ edge is considered, the Te M_45_ absorption corresponds to the *d→p* transition. Hence, with the same sign as the Cr L_3_ edge, which corresponds to the *p→d* transition, the measured sign of the XMCD signal at the Te M_5_ edge suggests an anti-parallel coupling between the Te 5*p* and Cr 3*d* spins.

Finally, by XAS and XMCD, we also investigated the Bi-site spin polarization for Cr_0.15_(Sb_0.9_Bi_0.1_)_1.85_Te_3_ (*T*_C_∼15K). The difference in the absorption spectra between left- and right-circular-polarized light (Fig. 3f,g) is more than 3 orders of magnitude smaller than the absorption intensity at the Bi N_45_ edges and Bi N_1_ edge (not shown), even under an external magnetic field of 1 T; this behaviour is in strong contrast to that occurred at the Sb- and Te-sites. These results clearly show that Bi contributes negligibly to the long-range magnetic order in the Cr-doped (Sb,Bi)_2_Te_3_ samples, and explain well the experimental fact that *T*_C_ decreases abruptly with increasing Bi concentration.

### *Ab initio* study of Cr-doping effect

**Figure 4:**
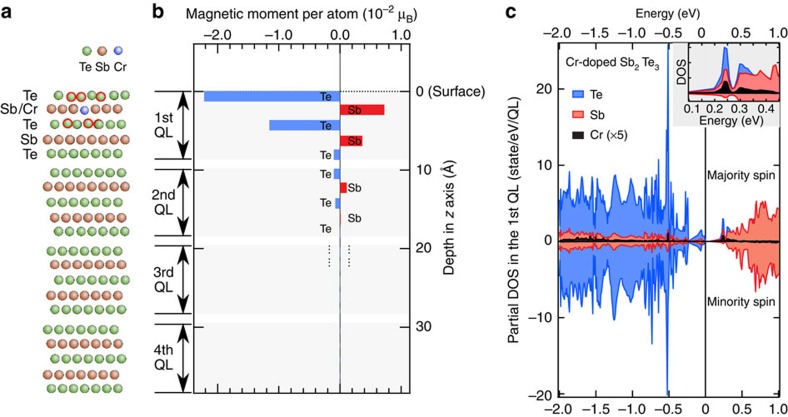
Calculated magnetic and electronic structures of Cr-doped Sb_2_Te_3_. (**a**) Relaxed atomic model of Sb_2_Te_3_ with one Sb atom replaced by Cr in the second atomic layer; (**b**) Atomic-layer-resolved magnetic moments induced by Cr in the host lattice of Cr-doped Sb_2_Te_3_; (**c**) Calculated partial density of states (DOS) in the 1st quintuple layer calculated with the model in **a**; inset, a magnified view for the electronic states involving *p–d* hybridization.

To explore the underlying nature of the long-range magnetic order induced by Cr-doping, we performed the first-principles calculations of the magnetic and electronic structures in the framework of density functional theory. In accordance with the previously reported result, that the Cr dopants favour the substitutional Sb site^13^, we considered a model of a Sb_2_Te_3_ slab comprising four quintuple layers (QLs) with a Sb atom in the second atomic layer from the surface replaced by a Cr atom (Fig. 4a). After full relaxation, the six nearest-neighbouring Te atoms slightly deviate from their original positions towards the substitutional Cr dopant (marked with red circles), suggesting a strong interaction of the Cr dopant with its surrounding Te atoms. The calculated magnetic moment for the Cr atom of 1.54 μ_B_, which is mainly contributed by the 3*d* derived electrons, is energetically favoured to align to the out-of-plane direction. The calculated valence electron number is 3.102 per Cr atom, which gives the valence state of Cr of 2.898+, slightly deviating from the 3+ states of Sb originally in the host lattice. This result explains the extra hole carriers induced when Sb is substituted by Cr as revealed by our photoelectron experiments (Fig. 1a,b). Importantly, the Sb and Te atoms in the host lattice, especially for those in the first QL with Cr dopant (see Fig. 4b), exhibit nonzero magnetic moments with magnitudes of 10^−2^ μ_B_ per atom, being 2 orders of magnitude smaller than the magnetic moment of Cr dopant, 1.54 μ_B_ per atom. This result is in good agreement with the experimental data, where the XMCD signal at Sb M_5_ edge for Cr_0.05_(Sb_0.9_Bi_0.1_)_1.95_Te_3_ is only 0.14% of the total absorption intensity, being 2 orders of magnitude smaller than that at Cr L_3_ edge (31.7%). Noting the signs of the magnetic moments, we find that the moments of Te- and Sb-layers are anti-parallel coupled with each other, where the magnetic moment of Sb-layers has the same sign as the Cr dopant; this is in good agreement with the experimental results. In addition, from proximity effects around the doped magnetic Cr atoms, although the magnetic moments for Sb and Te experience a rapid decay away from the Cr dopant into the bulk, the anti-parallel coupled moments of Te and Sb are found to penetrate into the second QL below the surface. Such results indicate that the magnetic coupling along the *c*-axis of the crystal is strong enough to form long-range magnetic order along the out-of-plane direction despite the existence of van der Waals gaps. Further computational analysis of the origin of the magnetic moments on the Sb- and Te-sites shows that their main contribution comes from the *p* states, thus providing the origin of the XMCD signals probed at the *d–p* transition edges for Sb and Te (Fig. 3a–e). Being similar to the traditional diluted magnetic semiconductors Mn-doped GaAs^24^,^25^, the *p* states are the main carriers in the Sb_2_Te_3_ systems. Thus, the magnetic moments determined by our XMCD experiments and our first-principles calculations reveal the nature of the carrier-mediated ferromagnetism in Cr-doped topological compounds. Such scenario can be understood as an analogy of the well-known diluted magnetic semiconductor, where the magnetic moment induced at As- (Ga-) site coupled anti-parallel (parallel) to the Mn 3*d* moment as reported by Keavney *et al.*^26^, and the polarized As valence holes mediate the ferromagnetic coupling between Mn ions in Ga_1−*x*_Mn_*x*_As. The spin-resolved partial density of states in the first QL (Fig. 4c), where the Cr dopant is situated, shows a strong hybridization between the Cr *d* states and the Te/Sb *p* states at 0.24 eV and 0.51 eV above and below the Fermi energy, respectively, especially for the states above the Fermi energy as illustrated by a zoom-in graph in the inset of Fig. 4c, which also evidences the crucial role of *p–d* hybridization. We also find a finite energy gap that appears in the calculated total density of states, which has its origin in the magnetism-induced time-reversal-symmetry breaking (Supplementary Fig. 8 and Supplementary Note 2).

Conjointly, the *p–d* exchange coupling strength is closely related to the energy splitting between the Cr 3*d* state and the Sb 5*p* (Bi 6*p*) states. Since the unoccupied states of Bi 6*p* are energetically much higher than those of the Sb 5*p*, the hybridization between the Cr 3*d* and Bi 6*p* states is negligible, thus yielding a negligible moment for Bi, in strong contrast to the situation for the Sb 5*p* state. This result unambiguously supports the absence of spin polarization on the Bi-site in the XMCD measurements (Fig. 3f,g), and explains the abrupt decrease of *T*_C_ when Sb is substituted by Bi.

## Discussion

From theory, the topological states of Sb_2_Te_3_, the parental material of Cr_x_(Sb_1−*y*_Bi_*y*_)_2−*x*_Te_3_, originate from the energy-band inversion between the *p* states of Sb and Te^5^,^6^,^7^. The XMCD intensity measured at the *d→p* transition edges (*M*_45_ edges) probed on the Sb- and Te-sites indicates a modification of the spin texture of the topological states when Cr is included that arises from time-reversal-symmetry breaking. This leads to the opening of the energy gap and reorientation of the spin along the perpendicular direction at the Dirac point. A direct observation of the spin texture for the present system by spin-resolved spectroscopy is strongly desired.

To conclude, we performed XMCD and photoemission spectroscopy experiments on the Cr-doped TI Cr_*x*_(Sb_1−*y*_Bi_*y*_)_2−*x*_Te_3_, which has been verified as a QAH system. We have shown that the *T*_C_ is enhanced by increasing the Cr-doping concentration. The magnetic moments with long-range order are probed not only for the Cr magnetic dopant but also on the Sb-sites; they are absent on the Bi-site. It is further found that the magnetic moment of Te favours an anti-parallel coupling with Cr dopants. These results clearly show that the long-range magnetic ordering is mediated by the *p* states of Sb and Te in the Cr-doped (Sb,Bi)_2_Te_3_ system through *p–d* hybridization, as supported by our theoretical calculations. The abrupt suppression of *T*_C_ with Bi substitution on the Sb site is ascribed to the fact that the Cr 3*d* states and the Bi 6*p* states are well separated energetically. This leads to a negligible *p–d* hybridization, which is a key factor in the formation of long-range magnetic order. These results give a deeper insight into the origin of the magnetism in TIs, and provide a way to manipulate the magnetism and quantum transport properties of a QAH system. We further revealed that proper tuning of the hybridization strength between the magnetic dopant and host carriers should be a promising way to elevate the *T*_C_ in magnetically doped TIs.

## Methods

### Crystal growth and characterization

The Cr-doped (Sb, Bi)_2_Te_3_ samples were grown by modified Bridgman method. First, high purity of Sb (99.999%), Bi (99.999%), Cr (99.99%) and Te (99.999%) powders were mixed and then sealed in evacuated quartz ampule. The mixed materials were initially heated to 900 °C and kept for 24 h, then slowly cooled to 550 °C within 48 h, followed by 72 h annealing at 550 °C. Finally, the crystals were naturally cooled to room temperature. The resultant sample crystals show shinny (0001) surface plane after cleavage. XRD measurements show clear diffraction peaks as indexed in Supplementary Fig. 1, and the presence of the secondary phase can be ruled out. The Cr-doped (Sb, Bi)_2_Te_3_ samples show clear ferromagnetic transition as temperature goes down in the *M*–*T* measurement by SQUID and XMCD (see main text). We also examined the magnetization as a function of magnetic field by SQUID, where the magnetic fields were applied perpendicular and parallel to the (0001) surface. Supplementary Fig. 2 reveals that the easy axis of the magnetization is along the *c*-axis of the crystal for both samples with different Bi concentrations. However, the saturated magnetization decreases when the Bi concentration increases from 0.1 to 0.3, which is in good agreement with the *M–H* curves measured by XMCD at Cr L_3_ edges as shown in Supplementary Fig. 7a. To further confirm the ferromagnetic order of our Cr-doped (Sb, Bi)_2_Te_3_ samples, we also performed XMCD measurement with remnant magnetization, and compared with the XMCD spectrum taken under external magnetic field (0.1 T). As shown in Supplementary Fig. 7b, a clear XMCD signal from Cr L_23_ edges is observed (grey circles), reproducing all of the spectral features that were revealed by XMCD data taken with 0.1 T field (black solid line). The relatively poor statistics of the spectrum at remnant magnetization is attributed to the narrow magnetic hysteresis loop and diluted concentration of Cr of the Cr-doped (Sb, Bi)_2_Te_3_ samples.

### XAS/XMCD experiments

XMCD is defined as the difference of the absorption rate between the left- and right-circularly polarized x-ray photons. When the sample is magnetized, the difference of the absorption intensities for differently polarized photon will be observed due to the selection rules, which states that the change of magnetic quantum number Δ*m* should be −1 or +1 depending on the circular polarization (Supplementary Fig. 4a). While the absorption energy depends on specific element, the XMCD method is thus element-selective by choosing the proper photon energy range. Technically, there are several methods to measure the absorption of X-ray photon. However, due to the rather small penetration depth of photon in the soft-x-ray region, one usually measures the fluorescence or the photocurrent, which are proportional to the absorption rate of the soft-x-ray. Supplementary Figure 4b illustrates the set-up for the measurement of photocurrent, namely the ‘TEY' method. Being limited by the penetration depth of soft-x-ray photons and the escaping length of the photoelectrons, the probing depth with the TEY method is limited at around a few tens of nanometres near the surface region. The XMCD experiments in this work were conducted in a TEY mode at the soft X-ray beamline BL23SU of SPring-8. The beamline is equipped with the twin-helical-undulator of in-vacuum type, which consists of two in-line helical undulators providing almost complete left- and right-circularly polarized x-ray, respectively. During the data acquisition, the polarization of x-ray is switched at every energy point in frequency of 1 Hz by five kicker magnets (see Supplementary Fig. 5). Such polarization switching at each energy point can ensure the identical sample condition for the measurements with different polarization, and thus XMCD data with excellent signal-to-noise ratio can be realized very efficiently (see ref. 27 for more details). All samples were cleaved *in situ* in an ultrahigh vacuum (better than 5 × 10^−8^ Pa) at room temperature, and then transferred to the liquid helium-cooled manipulator in the measurement chamber (better than 5 × 10^−9^ Pa) equipped with a superconducting magnet. The XAS and XMCD spectra were acquired in a TEY mode. All of the measured sample surfaces were confirmed to be oxidization-free through the experiments by measuring the oxygen 1*s* absorption edge before and after XAS/XMCD measurement of each sample. During the XMCD measurement, the Cr-doped (Sb,Bi)_2_Te_3_ samples were magnetized by a superconducting magnet. To eliminate the experimental errors that originate from the subtle difference between two undulators for left- and right-polarization, two XMCD spectra with reversed magnetization direction were recorded and averaged for each absorption edge. To perform quantitative analysis on the XMCD data, the experimentally obtained XAS and XMCD spectra have to be normalized. In the main text, to directly compare the magnitude of the XMCD signal at Cr *L*-edges and Sb *M*-edges, we normalized the intensity of the polarized XAS spectra at 595 eV to be 1 (see Supplementary Fig. 6a). The normalizing factor was then applied to the whole energy range for different absorption edges for each sample as shown in the Supplementary Fig. 6. The XMCD spectra (Supplementary Fig. 6b) were obtained by taking the difference of the normalized circular-polarized XAS, which then makes the comparison between different absorption edges reasonable.

### ARPES experiments

The ARPES measurement was performed with a hemispherical photoelectron analyser (R8000, VG-SCIENTA) at 70 K using a monochromized He Iα(21.2 eV) as excitation light source. The energy resolution of the ARPES measurement was set to 15 meV, and the vacuum was kept better than 1 × 10^−8^ Pa during the measurement. The calibration of the binding energy of photoelectron spectra was carefully carried out by measuring the Fermi edge of gold film, and fitting with the Fermi–Dirac distribution function.

### The first-principles calculation

In the first-principles calculations, the plane-wave basis method and the Perdew–Burke–Ernzerhof exchange correlation potential^28^ have been used as implemented in the VASP code^29^,^30^. In addition, the spin-orbit coupling is also included in all calculations. A 300-eV cut-off in the plane-wave expansion and a 4 × 4 × 1 Gamma *k*-grid were chosen to ensure that the calculations have an accuracy of 10^−5^ eV, and the internal coordinates of the large supercell (of size 3 × 3 × 1) with one of the Sb atoms substituted by a Cr magnetic dopant were optimized until forces on individual atoms became smaller than 0.005 eV Å^−1^ to obtain sufficient accuracy throughout the calculations.

## Additional information

**How to cite this article:** Ye, M. *et al.* Carrier-mediated ferromagnetism in the magnetic topological insulator Cr-doped (Sb,Bi)_2_Te_3_. *Nat. Commun.* 6:8913 doi: 10.1038/ncomms9913 (2015).

## Supplementary Material

Supplementary Information Supplementary Figures 1-8 and Supplementary Notes 1-2.
