# Supplementary material for: Carrier-mediated ferromagnetism in the magnetic topological insulator Cr-doped (Sb,Bi)2Te3
Source: Nat Commun. 2015 Nov 19;6:8913. doi: 10.1038/ncomms9913 (PMC4673827; doi:10.1038/ncomms9913)
Supplement: Supplementary Information — Supplementary Figures 1-8 and Supplementary Notes 1-2. [file ncomms9913-s1.pdf]

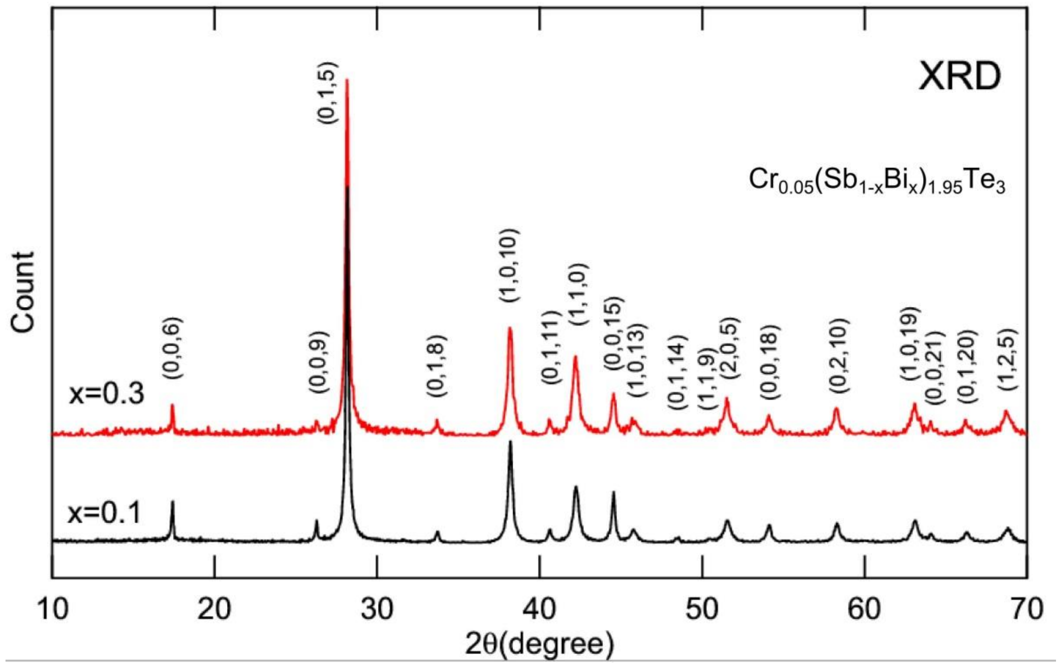

Supplementary Figure 1: Sample quality examined by X-ray diffraction (XRD) after doping. XRD results of $Cr_{0.05}(Sb_{1-x}Bi_x)_{1.95}Te_3$ for x = 0.1 and 0.3

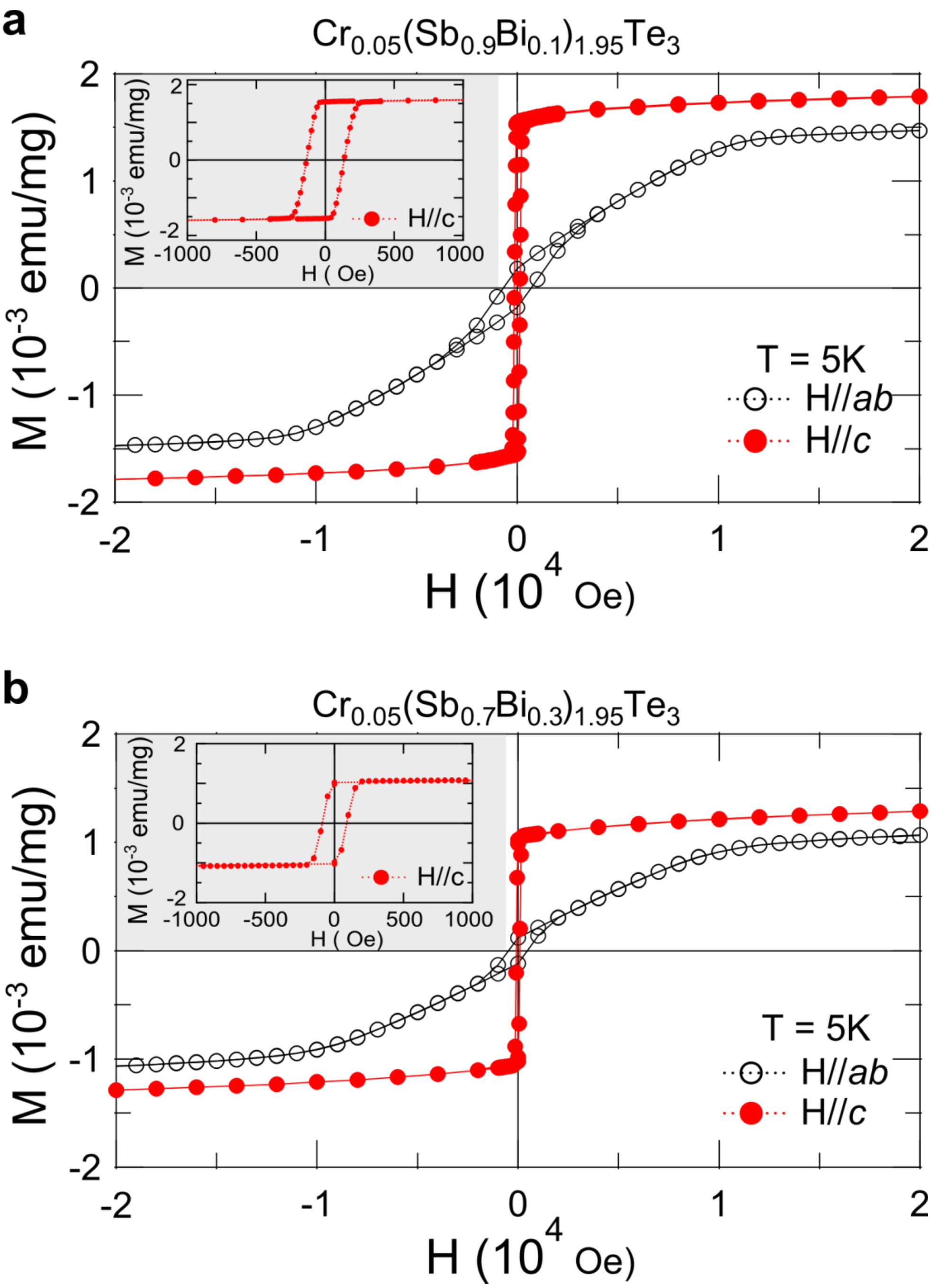

Supplementary Figure 2: Magnetization curves of $Cr_{0.05}(Sb_{1-x}Bi_x)_{1.95}Te_3$. (a) x=0.1 and (b) x=0.3 measured at 5K by superconducting quantum interference device. The magnetic fields are applied perpendicular (H//*c*) and parallel (H//*ab*) to the surface. Insets, magnified view in the vicinity of zero field for H//c, showing a clear hysteresis loop.

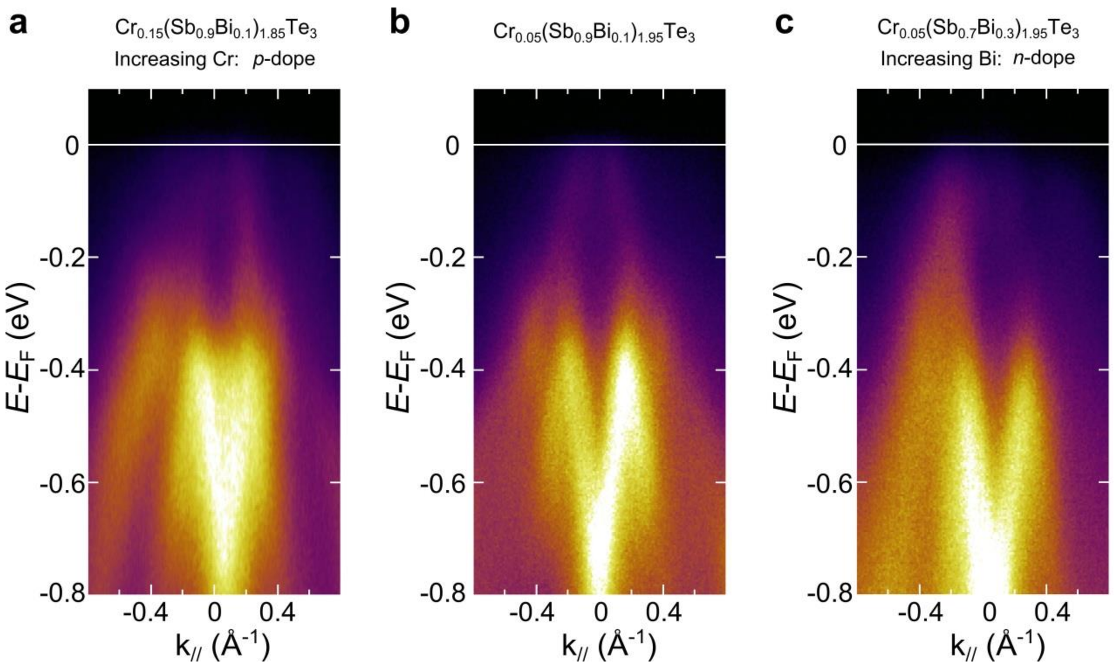

Supplementary Figure 3: Bi-doping effect. Angle-resolved photoelectron spectra taken along $\bar{\Gamma}$-$\bar{M}$ direction for (a) $Cr_{0.15}(Sb_{0.9}Bi_{0.1})_{1.85}Te_3$, (b) $Cr_{0.05}(Sb_{0.9}Bi_{0.1})_{1.95}Te_3$, (c) $Cr_{0.05}(Sb_{0.7}Bi_{0.3})_{1.95}Te_3$.

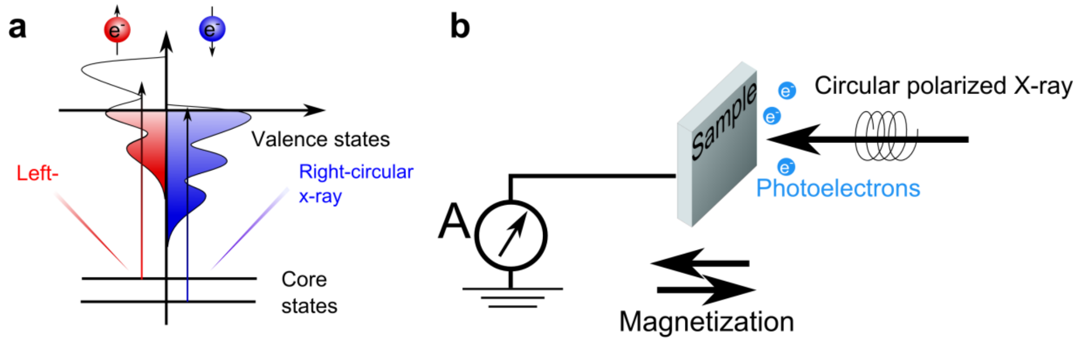

Supplementary Figure 4: Principle and setup of X-ray magnetic circular dichroism (XMCD) experiment. (a) Schematic of XMCD spectroscopy. (b) Schematic image of experimental setup for XMCD using total electron yield method.

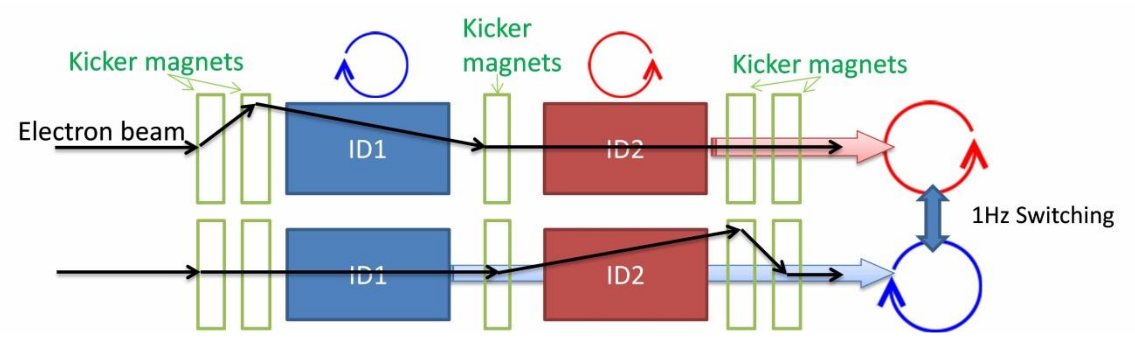

Supplementary Figure 5: Helicity switching of x-ray photons. Schematic drawing for the helicity switching of soft-x-ray by kicker magnets in the twin-helical-undulator.

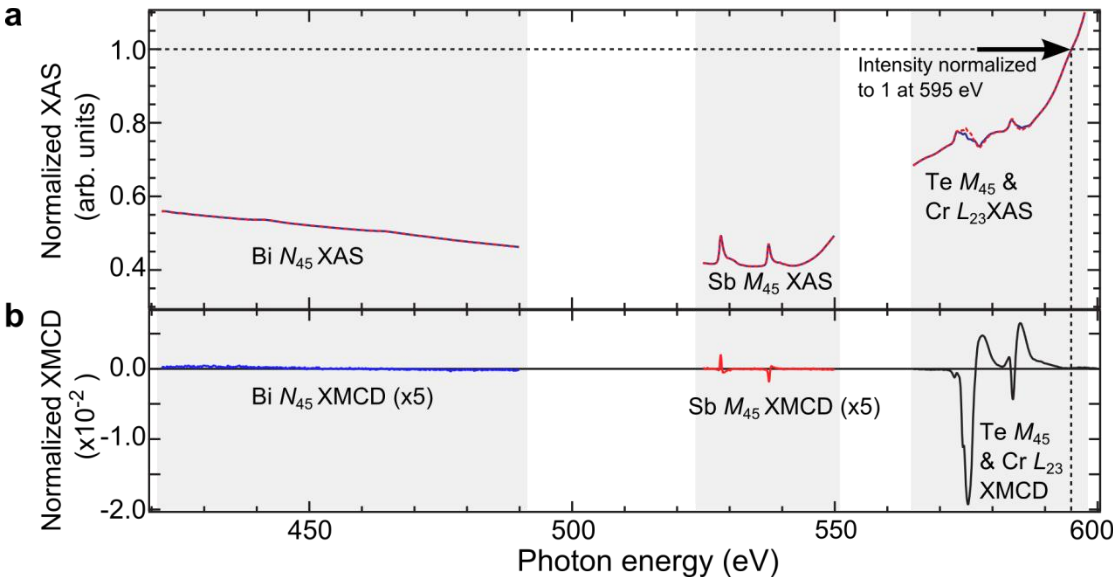

Supplementary Figure 6: Normalization process of the X-ray absorption spectroscopy (XAS) and X-ray magnetic circular dichroism (XMCD). (a) Normalized XAS spectra measured for $Cr_{0.15}(Sb_{0.9}Bi_{0.1})_{1.85}Te_3$ samples at 5K and 0.1T. (b) Normalized XMCD spectra for $Cr_{0.15}(Sb_{0.9}Bi_{0.1})_{1.85}Te_3$ samples at 5K and 0.1T.

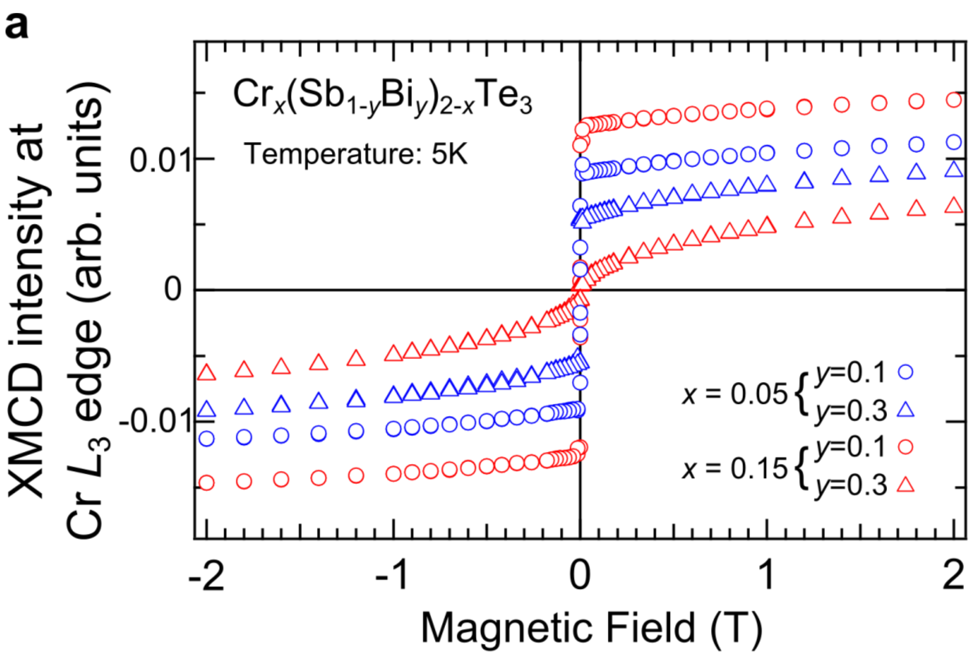

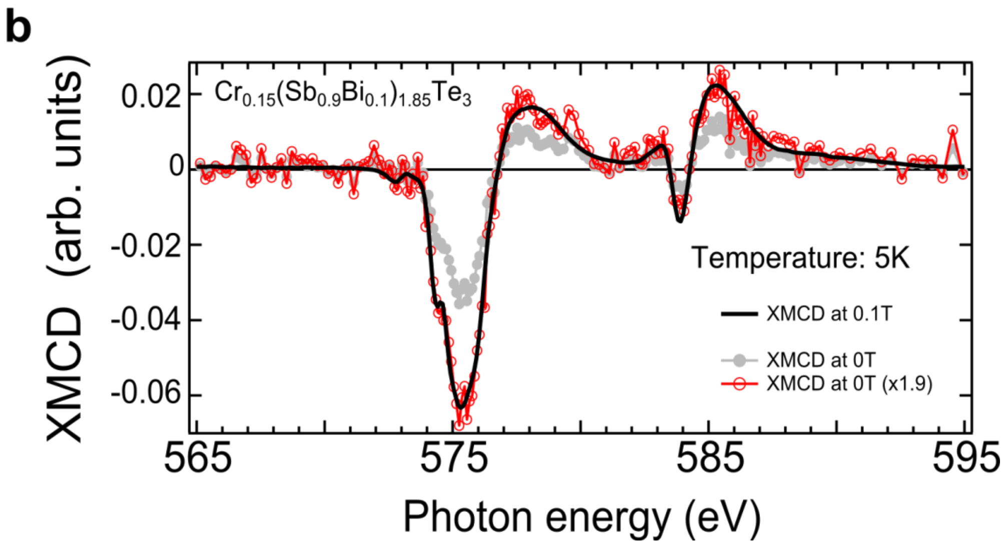

Supplementary Figure 7: Magnetization curves and remnant magnetization. (a) The whole set of magnetization curves as a function of magnetic field of Cr-doped (Sb,Bi)$_2$Te$_3$ samples measured at Cr L$_3$ edge with various Cr and Bi concentration. (b) X-ray magnetic circular dichroism spectra of Cr$_{0.15}$(Sb$_{0.9}$Bi$_{0.1}$)$_{1.85}$Te$_3$ measured with remnant magnetization (magnetic field: 0T; temperature 5K), compared with data taken under 0.1T external field.

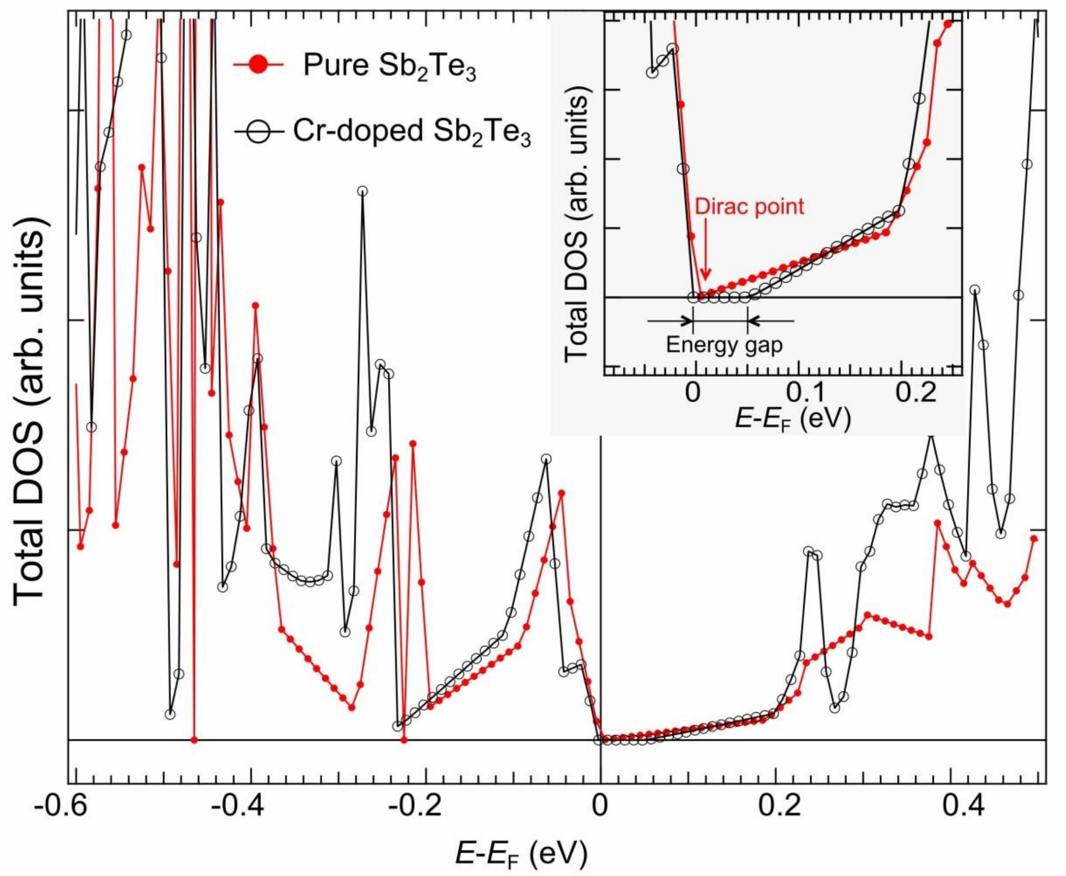

Supplementary Figure 8: Gap-opening at Dirac point. Comparison of calculated total density of states for Cr-doped and pure Sb_2Te_3 with 4 quintuple layers thickness. Inset, magnified view in the vicinity of Fermi energy.

Supplementary Note 1: Doping effect of Bi

In order to tune the chemical potential as close as possible to the Dirac point, Sb atoms are partially substituted by Bi. As shown in Supplementary Figure 3a, increasing Cr concentration leads to a slight increase of hole carriers, as discussed in the main text. Bi-substitution on the Sb-site leads to a global shift of valence band towards the higher binding energy (Supplementary Figure 3c). However, a significant modification of valence band dispersion is observed when 30% of Sb is substituted by Bi (Supplementary Figure 3c). The valence band dispersion with larger momentum (around 0.3$Å^{-1}$) protrudes towards the Fermi energy, showing an enhanced asymmetry with respect to the Γ point ($k_{//}$ = 0 $Å^{-1}$) due to a pronounced three-fold symmetry from the bulk electronic structures of Bi_2Te_3. This may result in an energetic overlap of surface and bulk states, which is a disadvantage in the device application based on the pure surface transportation with topologically non-trivial states.

Supplementary Note 2: Calculated energy-gap at Dirac points

Our calculated electronic structures also reveal an energy gap (~50 meV) in the vicinity of Dirac point as shown in Supplementary Figure 8 for the Cr-doped Sb_2Te_3 supercell model. In order to confirm the magnetic origin of the energy gap opening at the Dirac point revealed in our first-principles calculation, we have compared this result with undoped system with the same finite slab size and demonstrated that the perfect Dirac like surface states emerged in the bulk gap.
